# The Finsen Light Cure

**Published:** 1904-02

**Authors:** H. John Stewart

**Affiliations:** Chicago, Illinois


For Texas Medical Journal.

### The Finsen Light Cure.

BY H. JOHN STEWART, M. D., CHICAGO, ILLINOIS.

Having read and heard so much about the Finsen light treatment in the curing of disease, I decided in April of this year, to make a personal investigation to see and learn for myself if it was true that such diseases as lupus and rodent ulcer could be cured by light. I visited several institutions where the Finsen lamp was in operation. In Manchester, England, in the Salford Skin Hospital, they had a Finsen light department under the supervision of Prof. Brooke, who informed me they were unable to treat half the sufferers who applied for treatment, and they had solicited by public subscription $125,000 for the erection of a new hospital for skin diseases, where they would be able to enlarge the "light department" so at least two hundred people could be treated daily, as there were people on their waiting list whom they would be unable to treat, with their present facilities, for an indefinite time. Prof. Brooke was most enthusiastic over the wonderful results they were obtaining there.

I next visited the London General Hospital, of London, England, and found they were just completing an immense light department, that had been established by the present Queen of England, then Princess of Wales, in 1900, who presented the first lamp at that time, and it was found to be far too inadequate. She had just given a second lamp and Alfred Harmsworth had also given $50,000 for the perpetual endowment of another Finsen lamp in this department, and they were then building a platform to receive the king and queen, whom they expected to come June 11th to dedicate this new department, and even with these increased facilities, I was informed by Prof. Sequirey, there were patients on the waiting list who were unable to receive treatments.

I next visited the Light Institute at Copenhagen and found that all the statements that had been made regarding it were not in the least exaggerated. I had the pleasure of meeting and studying under Prof. Finsen himself and was extended every courtesy by Prof. Finsen and his assistants at this institution. He seemed very much pleased to describe in the minutest detail the apparatus, treatment, etc., and gave me a detailed history of the Finsen light.

The Finsen light is a large specially constructed arc lamp of 20,000 candle power, or twenty times stronger than an ordinary street light and uses from sixty to eighty amperes of current. This lamp burns a specially made carbon, which can only be procured at Copenhagen. In the upper holder is a large carbon, while a smaller one is used in the bottom holder; when properly adjusted for arcing a maximum number of violet and ultra violet rays are produced. The advantage of the Finsen lamp over others is in the greater number of violet rays produced. The Finsen lamp produces a much greater number of chemical rays than sunlight, as the atmosphere absorbs a large percentage of these rays. The light is so intense it is impossible to look at it with the naked eye and it is necessary for all the attendants and patients to wear dense smoked glasses while the lamp is in operation. An aluminum hood about two feet wide surrounds the lamp, which hood is fringed on its lower border with a deep crimson colored paper skirt to further aid in excluding the diffused light from the patients.

The concentrated rays are carried from the arc to the patients through four telescopic tubes, known as converging tubes, suspended at an angle of forty-five degrees, the tubes containing a series of rock crystal lenses so arranged that reservoirs for running water exists between them. By means of the water screen and rock crystal lenses, all rays but the violet are eliminated, and these rays are convergent and concentrated, thus vastly increasing the healing and bactericidal effects.

The heat from the original arc is so intense that to prevent cracking of the lenses and discomfort to the patients, a stream of cold water is kept constantly circulating through the reservoirs or water screens.

To further concentrate and cool the rays a compressor is provided which consists of two rock crystal lenses so arranged that a chamber for running water exists between them. This part of the apparatus is used to compress the affected area and make it bloodless during the treatment, thus facilitating deeper penetration. The Finsen arc light has been usd with marked success in curing many skin diseases, thought until this time incurable, especially lupus and rodent ulcer. During a period of six years the Finsen Medical Light Institute at Copenhagen has grown from a very small shed, where they were only able to treat one patient at a time, to a magnificent institution, where they are now treating three hundred people daily, and light institutes have been established in London, England; St. Petersburg, Russia; Paris, France; and Chicago, Illinois, where they are all carrying on a similar work to the parent institution.

It has been a popular belief that lupus was a very rare disease and common only in the northern countries, and although it was supposed there was no lupus in London, yet the hospitals are now treating 175 daily and the management was compelled to install two more lamps and build a separate department, so great has been the demand from people seeking relief. Lupus was considered very rare in the United States, but since the establishment of the Finsen Light Institute in Chicago, the author is informed, they have been taxed to their utmost capacity, and they, too, have found it necessary to increase their facilities as there are now patients on the waiting list who are not able to receive treatment. It seems but a question of a short time until light institutes will be established in every large city in America, because it has proven so efficacious in many other skin diseases besides lupus and rodent ulcer, such as acne, alopecia areata, localized eczema, chronic ulcers and naevus. The treatments are given while the patients recline on couches. By firm pressure with the compressors on the tissue to be treated, the blood is removed and more heat can be borne and deeper penetration produced. This compression has another important advantage in that the bacteriological effect is greater because it has been shown that the corpuscles absorb a considerable portion of the rays and thus prevent deep penetration.

The affected area is about ten inches from the distal end of the converging apparatus and the treatments, or seances as they are called, take about one hour daily in lupus and rodent ulcer, and in other skin diseases from ten to twenty minutes, depending upon each individual case.

The results attained have been hardly less than marvelous since from carefully compiled statistics covering a series of over 300

cases of lupus treated at the Finsen Institute an overwhelming percentage of cures and an insignificant number of failures is shown, and Prof. Finsen goes so far as to say that in lupus vulgaris cures can be obtained in 97 per cent. of cases, even where the whole face is involved. In these eight hundred patients, with ages ranging from 5 to 74 years, the average duration of disease was eleven years. This treatment has an advantage over the X-ray in that there is no danger of burning and consequent sloughing. With the light treatment we are dealing with a known quantity, while with the X-ray we have an unknown quantity of uncertain action.

The light treatment causes no pain; a red erythemathous spot and blister appears where the light is applied, and in five or six days the scab falls off and the ulcer is healed beneath, and the skin is left free from scar or cicatrix, but red, the redness, however, after a variable period fades and leaves the skin white and uncontracted, except where there has been a loss of tissue from the disease before treatment.

In conclusion, the author would state that the possibilities for the light treatment in the curing of diseases are still unknown, and believes in a limited time it will take an exalted position in the field of medicine and surgery.

